# Authors' Reply: Commentary on “Protecting User Privacy and Rights in Academic Data-Sharing Partnerships: Principles From a Pilot Program at Crisis Text Line”

**DOI:** 10.2196/59734

**Published:** 2025-01-22

**Authors:** Anthony R Pisani, Carlos Gallo, Madelyn S Gould, Nitya Kanuri, John E Marcotte, Brian Pascal, David Rousseau, Megan L Ranney, Bob Filbin, Shairi Turner

**Affiliations:** 1 Department of Psychiatry, Center for the Study and Prevention of Suicide University of Rochester Medical Center University of Rochester Rochester, NY United States; 2 Department of Pediatrics University of Rochester Medical Center University of Rochester Rochester, NY United States; 3 Northwestern University Evanston, IL United States; 4 Department of Psychiatry Columbia University New York, NY United States; 5 Department of Epidemiology Columbia University New York, NY United States; 6 Chartis San Francisco, CA United States; 7 University of Michigan Ann Arbor, MI United States; 8 Stanford University Palo Alto, CA United States; 9 Henry J Kaiser Family Foundation Menlo Park, CA United States; 10 Yale School of Public Health New Haven, CT United States; 11 Meta (United States) Menlo Park, CA United States; 12 Crisis Text Line New York, NY United States

**Keywords:** ethics, data sharing, digital data, text messaging, technology, cooperative behavior

## Abstract

We appreciate Reierson’s thoughtful commentary on our 2019 paper, which described our experiences, ethical process, judgment calls, and lessons from a 2016-2017 data-sharing pilot between Crisis Text Line and academic researchers. The commentary raises important questions about the ethical conduct of health research in the digital age, particularly regarding informed consent, potential conflicts of interest, and the protection of vulnerable populations. Our article focused specifically on the noncommercial use of Crisis Text Line data for research purposes, so we restrict our reply to points relevant to such usage. While we acknowledge the limitations of Crisis Text Line’s Terms of Service as a means of informing users about data sharing for research, we maintain that our guidelines were ethically sound and aligned with well-established practices for institutional review board (IRB) review and researcher training. We emphasize the critical role of IRBs in ensuring that research involving vulnerable populations, including minors, is conducted ethically and with appropriate safeguards. Regarding potential conflicts of interest, we argue that unpaid, nonfiduciary advisory board service for a nonprofit organization does not constitute a conflict requiring disclosure. The transparent nature of our collaboration with Crisis Text Line, as evidenced by the authorship and acknowledgments in our paper, further underscores our commitment to ethical research practices. We recognize the complexity and evolving nature of the challenges surrounding data-sharing partnerships in digital health research. As the field progresses, we remain committed to ongoing, transparent engagement and to refining best practices in collaboration with colleagues, stakeholders, and the public. Our response aims to provide clarity and context for the concerns raised in the commentary while reaffirming the integrity and value of our original work. Ultimately, we maintain that our paper contributed meaningfully to the ongoing discourse on ethical data sharing and laid the groundwork for future improvements in this critical area of digital health research.

## Introduction

Reierson’s commentary [1] on our 2019 article [2] highlights important and timely questions about the ethical conduct of health research in the digital age. Interactions with increasingly ubiquitous health-related digital platforms generate enormous amounts of data in which individual users, future users, and society have a vested interest [3]. We share Reierson’s concerns about how and under what conditions users ought to be informed about how data generated from their interactions are included in health and prevention research, including research that aims to prevent suicide. We published this paper to share with the field our experiences, ethical process, judgment calls, and lessons from a 2016-2017 data-sharing pilot, understanding that science and ethics advance through publication, critique, and refinement.

Our article explicitly pertains to “noncommercial use of data” for the purposes of research and evaluation. The article neither addresses nor endorses use of Crisis Text Line data for commercial purposes. No members of the Crisis Text Line data advisory board that advised on the data-sharing pilot our article described served in any capacity with Loris.ai. Given that Reierson’s commentary on the commercial use of data and Loris.ai is outside the scope of our paper and anachronistic to the data pilot we reported on, we focus our response on his in-scope feedback. The commentary also questions common features and norms in human subject research (such as the definition of a research subject and the role of institutional review boards). The adequacy of well-established ethical frameworks and review processes is always worthy of review, but we will focus here on those questions relevant to our specific conduct.

With respect to including deidentified Crisis Text Line data in research, the commentary raised concern about: (1) the Terms of Service that Crisis Text Line used to inform users of data-sharing policies with respect to research and (2) potential undisclosed conflicts of interest in the author group.

## Crisis Text Line Terms of Service and Consent

Regarding the concerns raised about Crisis Text Line’s Terms of Service (Terms) with respect to research, we appreciate the limitations pointed out by Mr Reierson. At the time the paper was published, we considered the Terms to be an appropriate means by which to “inform users in an *unobtrusive way* that anonymized data are shared with select research partners.” We agree that the Terms could have been simplified with respect to research, and we understand that Crisis Text Line has since improved them.

It is important for the general public to understand that any use of Crisis Text Line data for research must first be reviewed by a researcher’s university institutional review board (IRB)—an independent committee that reviews the research protocol, including the adequacy of the consent process and the protection of vulnerable populations, such as minors. This review process is part of the context in which our guidelines were offered. Furthermore, all researchers undergo Human Research Protection Training, which emphasizes the Belmont Report’s ethical principles of respect for person, beneficence, and justice, with particular attention to the protection of vulnerable populations. IRBs often consider secondary analyses of data without access to identifying information as not meeting the definition of human subjects research according to the federal definition 45 CFR 46.102(f).

Thus, the authors of the article still conclude that what we wrote at the time adheres to ethical standards of research, even while we welcome retrospective critique of the recommendations we generated at the time. Furthermore, we believed—and continue to believe—that it is not just acceptable, but is imperative, for academic researchers to engage with technology companies to create methods and means for research that both improve the protections of people engaging with that technology and facilitate the use of the generated data for good rather than for harm.

## Potential Undisclosed Conflicts of Interest

Regarding questions raised about potential conflicts of interest, it should be noted that Crisis Text Line is a nonprofit organization, and no one on the Crisis Text Line data advisory board had a role in or even knew about Loris.ai, which was founded after the data-sharing pilot took place. We did not and do not consider volunteer nonfiduciary participation in the Crisis Text Line advisory board as a “conflict of interest,” and none of the authors had any other relationships or funding from Crisis Text Line at the time this article was written and published. The Crisis Text Line advisory boards convened volunteer experts to provide independent advice, an extremely common practice.

- Members of the Crisis Text Line advisory boards are unpaid and receive no other tangible or intangible benefit from their participation. They volunteer a small amount of time to provide input and expertise, hopefully to the benefit of the nonprofit organization and the people it serves.
- Crisis Text Line has no undue influence on advisory board members’ feedback or behavior. Advisory board members are sufficiently independent from Crisis Text Line to be able to provide critical feedback—both positive and negative—on Crisis Text Line’s operations.
- Our advisory board had no fiduciary responsibilities to Crisis Text Line.

In studying the Committee on Publication Ethics’ (COPE) guidelines, JMIR Publications’ policy, and other publicly available ethical statements, we could not find any indication that a relationship of this type would be considered a “vested monetary interest” or a “conflict of interest.” *[Editorial Note:While we found no intent to deceive on the part of the authors, we disagree with this interpretation; see corrigendum to Pisani et al’s 2019 publication [4]]*. We should note that our author group appreciated the opportunity to consider this question. Most agreed that, while not required nor customary, there would be no harm—and could be some potential benefit—in disclosing unpaid advisory board service, and some of us will choose to do so in the future (eg,[5]).

Further, far from being “undisclosed,” the participation and collaboration of members of the author group with Crisis Text Line in documenting the results of their pilot academic data-sharing program was a key feature of the article.

- Crisis Text Line employees were listed as authors along with their affiliation.
- An appendix was provided listing members of the data ethics committee, which included several of the authors.
- The paper is manifestly the result of collaboration with Crisis Text Line—academics and nonprofit leaders transparently described the ups and downs of a pilot program aimed at protecting privacy, conducting research ethically, and contributing to science and society.

## Summary

In summary, we share Mr Reierson’s concern about ethical use of data and appreciate his thoughtful interaction with our 2019 paper. Our article addressed only noncommercial use of data. We see Mr Reierson’s arguments about Terms of Service and consent as welcome and substantive critiques rather than ethical concerns about our 2019 paper. We stand by our work and recommendations as ethically sound, and we highlight that we wrote this publication to advance discourse and future ethical guidelines. Finally, unpaid, nonfiduciary advisory board service to a nonprofit organization by independent academic experts does not constitute a conflict of interest, although we see the benefit of listing such service in certain circumstances in the future.

We remain committed to ongoing, constructive scientific dialog and to refining best practices in collaboration with interested parties and the public. Working together, we can best realize the benefits of research while upholding high standards of ethics, privacy, and trust.

Note: The following authors of the original publication could not be reached or were unavailable to participate in this Authors’ Reply: Lisa Soleymani Lehmann, Robert Levine (deceased), and Shirley Yen.

## Abbreviations

COPE: Committee on Publication Ethics
IRB: institutional review board
